# Rudimentary Sympathy in Preverbal Infants: Preference for Others in Distress

**DOI:** 10.1371/journal.pone.0065292

**Published:** 2013-06-12

**Authors:** Yasuhiro Kanakogi, Yuko Okumura, Yasuyuki Inoue, Michiteru Kitazaki, Shoji Itakura

**Affiliations:** 1 Department of Psychology, Graduate School of Letters, Kyoto University, Yoshidahonmachi, Sakyo-ku, Kyoto, Japan; 2 Department of Computer Science and Engineering, Toyohashi University of Technology, Tempaku-cho, Toyohashi, Japan; Goldsmiths, University of London, United Kingdom

## Abstract

Despite its essential role in human coexistence, the developmental origins and progression of sympathy in infancy are not yet fully understood. We show that preverbal 10-month-olds manifest sympathetic responses, evinced in their preference for attacked others according to their evaluations of the respective roles of victim, aggressor, and neutral party. In Experiment 1, infants viewing an aggressive social interaction between a victim and an aggressor exhibited preference for the victim. In Experiment 2, when comparing the victim and the aggressor to a neutral object, infants preferred the victim and avoided the aggressor. These findings indicate that 10-month-olds not only evaluate the roles of victims and aggressors in interactions but also show rudimentary sympathy toward others in distress based on that evaluation. This simple preference may function as a foundation for full-fledged sympathetic behavior later on.

## Introduction

Sympathy, or the feeling of concern for others, plays a crucial role in human social relationships and constitutes one of the most important components of human coexistence. For centuries, philosophers have offered penetrating insights into its nature [1], and even now, it is the subject of expansive debates across multiple disciplines. However, despite its important role, its developmental origins and progression in infancy are not yet fully understood.

Researchers have suggested that even newborns respond to others in distress by resonating with others’ emotional states through mechanisms such as emotional contagion (e.g., crying when others cry [2], [3]) but that true other-orientation does not develop until the second year of life, when infants can differentiate between self and others (e.g., mirror self-recognition [4], [5]). Developmental studies tend to agree that sympathetic concern for others emerges around the age of 18 months [6], [7], and sympathetic response for others (e.g., comforting) develops over the second year of life [8], [9]. This behavior evolves rapidly, with 3-year-olds intervening to protect victims from an aggressor [10]. However, to the best of our knowledge, no study has investigated sympathetic behavior during the developmental period between the emergence of the sympathetic response for others in newborns and clear concern for others in toddlers. In an attempt to fill this gap, the present study explored whether preverbal infants show rudimentary sympathy for others.

It has been demonstrated that preverbal infants have well-developed socio-cognitive capacities before their second year of life [11]–[13]: for example, infants in their first year can discriminate between positive and negative interactions (hitting) in geometric figures [11]. In addition, infants in the second half of their first year showed a preference for or avoidance of characters who previously engaged in helping or hindering behavior, respectively [12]. In this study, when infants evaluated the hindering behavior, they required the notion that blocking (hitting) is bad behavior. Even in a more controlled experiment [14], infants regarded such hitting interactions as negative. Combined with the findings that infants begin to understand causal agency in the second half of the first year [15]–[17], these studies raise the possibility that infants have some (implicit or explicit) knowledge that hitting leads to the distress of the attacked others. Considering these studies, preverbal infants may possess the cognitive abilities necessary for showing a sympathetic response toward attacked others. In addition, de Wall has reported that implicit, automatic responses (e.g., approach) toward distressed others are often observed in primates; this is referred to as “preconcern” [18], [19]. According to his theory, organisms are naturally endowed with such responsiveness, which functions as a simple behavioral rule: “If you feel another’s pain, get over there and make contact.” Taken together, if infants watched an aggressive interaction, such as one agent hitting another, then they would show an automatic response (e.g., gaze and approach) toward the victim.

We used a simple geometric animation to initiate this rudimentary and automatic sympathetic response in infants; the use of such animated figures has been well established in many infant studies. Infants in their first year of life attribute goals and intentionality to geometric figures [20].Moreover, they can attribute disposition [21], valence [12], and social dominance [22], [23] to figures based on previous interactions among them. To investigate infant automatic response toward victims in an animated sequence, we used preferential-looking methods that examined visual preference [24] and forced-choice methods whereby infants could express preferences through reaching behavior [12]–[14]. In the present experiments, we hypothesize that if preverbal infants feel rudimentary sympathy for attacked others, they should manifest an automatic approach response toward the victim in third-party affiliation situations. Specifically, we hypothesize that after observing aggressor-victim interactions, infants will prefer victims to aggressors.

## Experiment 1

In Experiment 1, we tested whether preverbal 10-month-olds prefer victims to aggressors after observing aggressor-victim interactions (Aggressive Interaction [AI] condition). To confirm that the aggressive interaction between the two figures affected infants’ preferences, a control condition (No Interaction [NI] condition) was established in which the two figures appeared to move independently and without any contact.

### Methods

#### Participants

Forty 10-month-olds (20 male, 20 female; mean age = 301 days, range = 288–314 days) were randomly assigned to either the AI (*n* = 20) or NI conditions (*n* = 20). Eight additional infants were tested but not included in the final sample because of fussiness (*n = *2) or a failure to meet the inclusion criterion (*n = *6), which was reaching for or grasping a single object within 45 s of it being presented.

#### Stimuli and procedure

Infants were seated on their parents’ laps in a darkened room; they faced a 32-inch TV monitor that presented a video with animations of geometric objects. Parents were instructed not to talk, interact, or interfere with their infants during the experiment. The viewing distance between the monitor and the infant was approximately 60 cm.

The animated stimuli were created using Poser 6.0 (e frontier Inc.). The animated stimuli were presented on a monitor in the observation room via a remote laptop computer controlled by an experimenter. Adobe Flash CS3 Professional (Adobe Systems Inc.) controlled the animated stimuli. Videos of infant eye movements and the corresponding stimuli were viewed simultaneously as split and inverted images using a Mutech inversion memory unit (MVF-120) and a Houei Multi viewer (MV-40F). These videos were recorded for offline coding.

**Figure 1:**
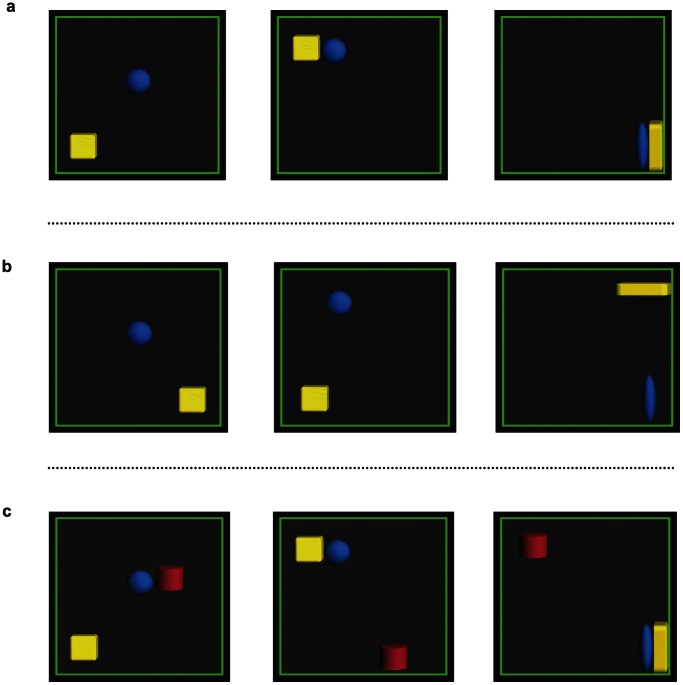
Selected frames from the movie stimuli in each experiment. Figure (a) shows the animated stimuli of the Aggressive Interaction (AI) condition in Experiments 1 and 2, in which one geometric figure crashes into the other. Figure (b) shows the animated stimuli of the No Interaction (NI) condition in Experiment 1, in which the two geometric figures moved independently and without contact. Figure (c) shows the animated stimuli in Experiment 2, in which two geometric figures interact in the same way as in (a), but the third figure moves independently.

In the familiarization phase, infants watched an animated sequence six times. The familiarization sequence showed a blue ball and a yellow cube moving across a black background within a green enclosure. During AI familiarization trials, the blue ball chased the yellow cube and hit it seven times during each trial, violently attacking and crushing the yellow cube at the end of each sequence (Fig. 1a and Video S1). In the NI condition, we changed the position of the victim figure such that the two figures appeared to move independently and without any contact (Fig. 1b and Video S2). Familiarization events in each condition presented two alternating examples (see another version of the aggressive interaction in Video S3). Movement speed, momentum, and extent of deformation were identical for the geometric figures in each condition. Each animated sequence lasted 20 s. Attractive animated clips with sound were played between trials to keep infant attention focused on the monitor. The roles of aggressor and victim for the two geometric figures were counterbalanced across participants.

During the test phase, infants were presented with a video showing the two, now static, geometric figures side by side against a black background for 30 s, to measure their preferences for each figure. In a subsequent choice task, an experimenter (blinded to the roles of the two geometric figures) presented two real objects on the desk in front of an infant and encouraged him or her to choose between a blue ball 6.5 cm in diameter and a yellow cube with 6.5 cm sides, corresponding to the animated geometric figures. The distance between the two objects was 30 cm. The presentation position of the two objects (left or right) was counterbalanced across participants.

#### Data analysis

The time that infants spent looking at each of the animated stimuli was recorded during the familiarization trials, as well as the time that each infant spent looking at whichever static geometric figure or figures during the 30-s preferential-looking test trial. Looking times for each infant were measured offline at a rate of 30 frames per second by two trained research assistants who were blind to experimental conditions. One assistant coded all participants, while the other independently coded a random 20% sample of participants in each condition. The two coders reached 91% agreement on the preferential-looking test trial for each of the looking-time frames.

After confirming that an infant had looked at both objects prior to responding in the choice test, preference for the objects was measured by recording which of the two objects the infant reached for or grasped first. To be included in the analyses, the infant had to reach for or grasp a single object (not two objects) within 45 s of the object presentation.

#### Ethics statement

The research was approved by the ethics review board at the Department of Psychology, Kyoto University. All infants participated with written informed consent from their parents.

## Results and Discussion

The two coders reached 96% agreement on looking time in the familiarization trials. Regarding the mean looking times, there was no significant difference between the AI and NI conditions (AI mean = 17.5 s, NI mean = 16.9 s, *t*(38) = 1.026, *p* = 0.312, *d* = 0.33).

**Figure 2:**
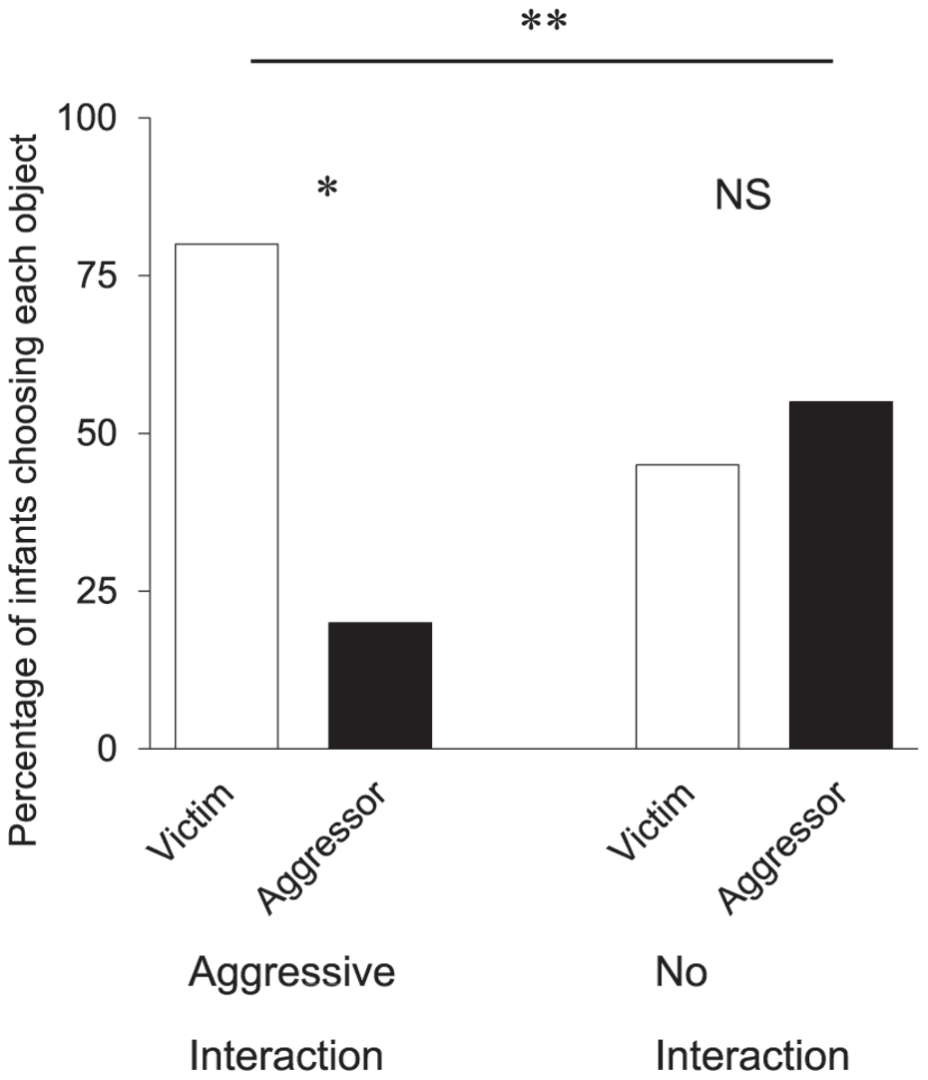
The results of the choice task in Experiment 1. This figure shows the percentage of infants who chose each object (Experiments 1, *n* = 20 in each condition). Single asterisks indicate statistical significance, one-tailed, *p*<.05. Double asterisks indicate statistical significance, two-tailed, *p*<.05. NS indicates not significant.

In the test phase, the looking-time measurements were analyzed using a mixed factorial analysis of variance with role (victim versus aggressor) as the within-participants factor and nature of interaction (AI versus NI) as the between-participants factor. We observed no significant main effects for role, *F*(1, 38) = 0.733, *p* = .397, η_p_^2^ = .019, or nature of interaction, *F*(1, 38) <0.01, *p* = .991, η_p_^2^<.001, nor was there a significant interaction between these two factors, *F*(1, 38) = 0.01, *p* = .920, η_p_^2^<.001. This null result indicates that infants did not preferentially look at either the victim or the aggressor in either interaction condition (AI: mean_victim_ = 8.2 s, mean_aggressor_ = 8.3 s; NI: mean_victim_ = 7.7 s, mean_aggressor_ = 7.6 s). In contrast, the choice measure revealed that infants robustly chose the victim in the AI condition (16 of 20 infants, binomial test, two-tailed, *p* = .012) but not in the NI condition (9 of 20 infants, *p* = .824). These outcomes reflect a significant difference between the two conditions (Fisher’s exact test, two-tailed, *p = *.048; see Fig. 2).

We found that infants preferentially reached for the victim over the aggressor in the AI condition but not in the NI condition, indicating that infants formed different evaluations for figures based on the nature of their previous interactions and preferred others in distress. These results cannot be explained by low-level perceptual interpretations, at least such as movement speed, kinetic momentum, and deformation, because they were the same for the two figures. In addition, there was no difference in looking time during the familiarization trials for both conditions. Thus, infant preference can confidently be attributed to differential interactions between the geometric figures in the two conditions. Therefore, our findings suggest that 10-month-old infants show sympathetic responses toward attacked others.

The lack of correspondence between looking-time and choice behavior may be attributable to insufficient exposure time in the familiarization trials, as infants’ looking-time preferences sometimes vary because of factors such as stimulus complexity and duration of exposure (e.g., [25]). As another possibility for this lack of correspondence is that while some infants might prefer the victim, others might look longer at aggressors because they pose a threat. Indeed, 9 of the 16 infants who chose the victim preferentially looked at the victim, and the remaining 7 preferentially looked at the aggressor. However, when choosing the character, they might have consistently reached for the character that they wanted to approach, because they had to make contact with that character. Although previous studies have shown correspondence between preferential looking and choice behavior [24], [26], another recent study has reported a discrepancy in this correspondence [27], much like in the present study. A third possibility is that the difference between this and previous studies may be due to the different preferential looking methods (the use of actual objects versus virtual objects displayed on a screen).

## Experiment 2

Experiment 1 demonstrated that infants behaviorally preferred victims. However, it is conceivable that the infants acted not out of sympathetic feeling for the victim but out of a desire to avoid the aggressor. We examined this possibility in Experiment 2 by repeating Experiment 1 with an added, neutral object during familiarization trials. Thus, each familiarization video included three objects: a victim, an aggressor, and a neutral. In the test phase, infant selection of a neutral object or a victim object (Neutral-Victim [NV] condition), or a neutral object or an aggressor object (Neutral-Aggressor [NA] condition) was assessed.

### Methods

#### Participants

Twenty-four 10-month-olds (12 male, 12 female; mean age = 297 days, range = 285–312 days) were randomly assigned to either the NV (*n* = 12) or the NA conditions (*n* = 12) group. Seven additional infants were tested but not included in the final sample because they failed to meet the inclusion criterion, which was the same as Experiment 1.

#### Stimuli and procedure

The materials and procedure were identical to those in Experiment 1, except that (1) neutral geometric figures and objects (a red cylinder, 6.5 cm in diameter and length) were added in the familiarization and test phases, respectively (see Fig. 1c and Video S4) and (2) we did not conduct a preferential-looking task in the test phase, although we increased the number of familiarization trials from six to eight.

In Experiment 2, a third, neutral geometric figure moved independently of the other two figures. The neutral figure had the same movement speed, momentum, and extent of deformation as the other figures. However, to emphasize the independence of the third neutral figure, the timing of its deformation was not synchronized with that of the other two objects. The position of the third figure was determined by inverting the average coordinate axis between the other figures (see Fig. 1c).

#### Data analysis

Infant looking time at each of the animated stimuli was calculated during the familiarization trials. In addition, to investigate the differences in perceptual exposure between the neutral object and the other two objects, infant gaze-shift frequency between the neutral and victim/aggressor objects was recorded. In the choice measure, the analysis inclusion criterion was the same as in Experiment 1.

## Results and Discussion

In the familiarization trials, we used the same coding method as in Experiment 1, and the two coders reached 97% agreement on the looking time in each of the familiarization trials. Regarding the mean looking times, there was no significant difference between the NV and NA conditions (NV mean = 15.6 s, NA mean = 17.2 s, *t*(22) = 1.607, *p* = 0.122, *d* = 0.66). In addition, regarding the mean gaze-shift frequencies from the neutral object to the other objects (or vice versa), we observed no significant difference between the NV and NA conditions (mean NV gaze shifts = 8.4; mean NA gaze shifts = 10.0, *t*(22) = 1.305, *p* = 0.205, *d* = 0.53).

**Figure 3:**
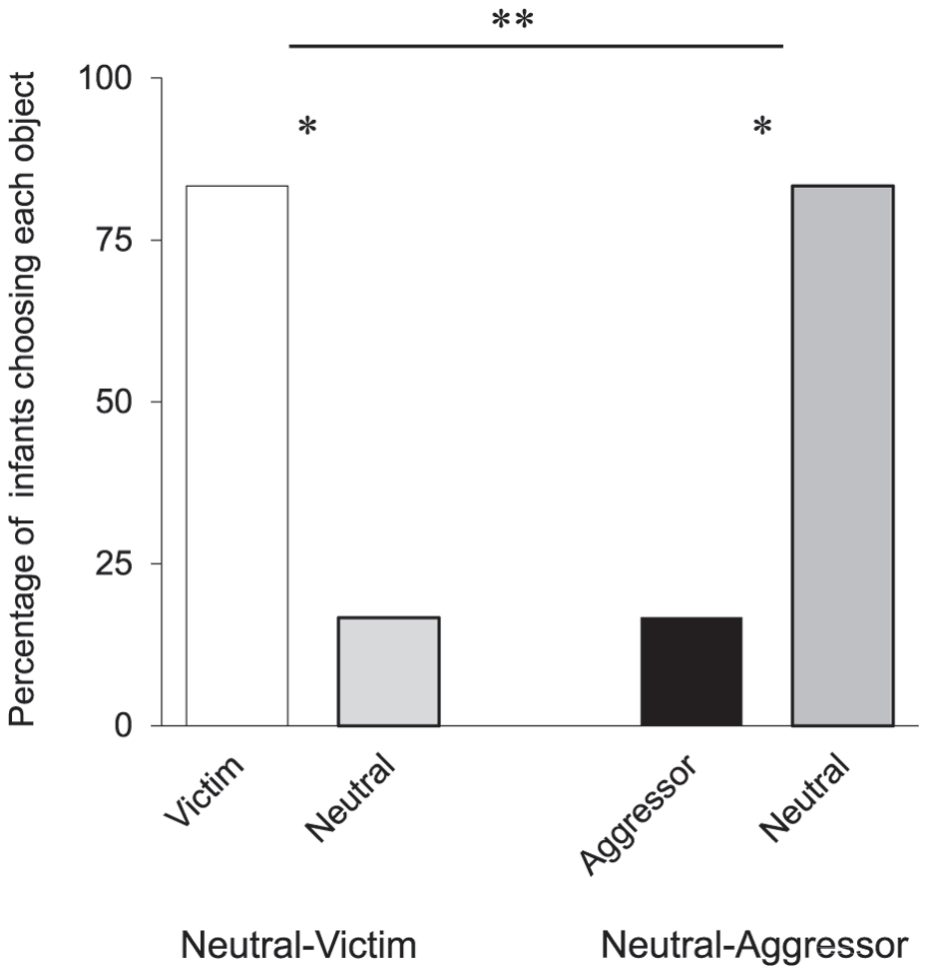
The results of the choice task in Experiment 2. This figure shows the percentage of infants who chose each object (Experiments 2, *n* = 12 in each condition). Single asterisks indicate statistical significance, one-tailed, *p*<.05. Double asterisks indicate statistical significance, two-tailed, *p*<.05. NS indicates not significant.

The results from the test phase show that infants responded differently to neutral objects in the choice task depending on whether they were paired with victim or aggressor objects (Fisher’s exact test, two-tailed, *p = *.003; see Fig. 3). Infants in the NV condition robustly chose the victim (10 of 12 infants, binomial test, two-tailed, *p* = .039), while infants in the NA condition robustly chose the neutral object (10 of 12 infants, *p* = .039). These results cannot be attributed to differences in perceptual exposure between neutral objects and other objects, because infants frequently shifted their gaze back and forth between the neutral object and the other objects throughout the familiarization phase. In addition, there was no difference in looking time during the familiarization trials for both conditions. Therefore, infants’ subsequent choice behaviors were most likely related to different evaluations of the objects during the two choice conditions. The different object-pairing results show that infants preferred victims and avoided aggressors. This finding indicates that preference for victims in Experiment 1 cannot be explained just by the desire to avoid aggressors; instead, it appears that infants evaluate the respective roles of victim and aggressor in interactions between the two.

### Conclusions

In investigating sympathetic behavior in preverbal 10-month-old infants, we demonstrated that they preferentially reached for victims as opposed to aggressors and neutral objects after observing third-party social interactions involving aggression. These findings indicate that preverbal infants show a sympathetic response toward attacked others who displayed no distress, suggesting that rudimentary sympathy for others based on an evaluation that is beyond merely a response to distressed others through emotional contagion [2], [3] occurs earlier in development than previously assumed. Although emotional contagion may be the mechanism of this sympathetic response [18], [19], our results cannot be explained solely by emotional contagion, because victims did not express emotional signals and because infants responded after the fact on the basis of their evaluations of third-party interactions, abstracted from the actions of geometric figures. Indeed, one recent study has demonstrated that toddlers show sympathetic concern for distressed others in the absence of a distressed emotional cue [7].

So what are the socio-cognitive processes behind infants’ evaluations, and how do they work to produce such sympathetic responses? To evaluate each character in our task, infants needed to understand who attacked and who was attacked. This cognitive process requires an understanding of the goal directedness of the agents and the causal relationship between them. Previous studies demonstrated that, after six months of age, infants begin to understand goal directedness [28], [29] and causal agency [15]–[17]. This representation of agency constitutes an important aspect of human cognition and is a key attribute in human ontogeny [30]. We suspect that because infants discriminated between the roles of the geometric figures, they had some understanding of agency; thus, sympathetic responses may rest on this sense of agency. Moreover, to evaluate the characters, infants in our study needed to regard the aggressive interactions as negative events. Recent studies have demonstrated that 10-month-olds regard such hitting interactions as negative [14]. This negative evaluation for hitting interactions might derive from everyday interactions with parents and siblings (e.g., experiencing being hit and watching others being hit). Alternatively, as in a series of studies by Hamlin [12], [13], [24], infants might acquire the cognitive ability necessary for this evaluation earlier in their development. With any possibility, it is possible that by combining many already-developed cognitive abilities, infants begin to evaluate the identities of the victim and aggressor based on their interaction, which results in a sympathetic response toward the victim.

It is perhaps unclear whether this preference for the victim in our task is derived from rudimentary sympathy. In fact, in our experiment, infants did not manifest clear concerns or attempt to comfort the victim. Nevertheless, previous studies have reported that in early infancy, infants have well-developed socio-cognitive abilities [11]–[14], and even rudimentary empathy [18], [19], making it plausible that this preference is derived from sympathetic feelings. That said, it is likely that this preference for the victim is a rudimentary form of sympathy. By using electroencephalography data [31] and an index of physiological response such as stress and pupil dilation [32] in our task, we might be able to provide additional evidence that this preference for the victim is derived from sympathetic feelings.

Although the sympathetic disposition reported here is not full-fledged, a basic preference for the victim might function as a foundation for more mature sympathetic behaviors, such as the sympathetic concerns for distressed others that emerge later in development [6], [7]. This sympathetic disposition may make young children more likely to attend to and approach others in trouble. However, presently, it is unknown whether this sympathetic disposition is related to full-fledged sympathetic behavior which emerges later in development. Further studies are needed to confirm the relationship between this disposition and the sympathetic behavior observed in previous studies.

Recently, there is an ongoing debate about how to interpret the results of this type of experiment. Scarf et al. demonstrated that controlling for low-level perceptual information–such as whether the acted character bounces–affects infant choice behaviors, casting doubt on the more rich interpretation that social evaluation influences infant behavior [14]. To address this, we attempted to make the presented stimuli in the experimental condition as simple as possible by removing extraneous information and making the movement speed, kinetic momentum, and deformation constant between the agents. However, in the control condition, to ensure that this kind of low-level perceptual information was the same as that in the experimental condition, some elements of the interaction between the two agents could not be controlled (e.g., the characters were crushed in different directions between the conditions). However, to eliminate any effect of interaction, such control procedures were deemed appropriate, because it is reasonable to believe that infants might have observed an interaction even if the two characters had had no contact. Thus, although not all possible interaction elements between the two agents were controlled for in the control condition, we believe that the results of our experiments can withstand such criticism.

In conclusion, by the age of 10 months, preverbal infants prefer victims to aggressors and neutral objects when evaluating third-party social interactions. This finding indicates that preverbal infants show rudimentary sympathy toward others based on their evaluation of characters’ interactions. Discovering how this disposition emerges throughout development and contributes to later sympathetic behavior requires additional research.

## Supporting Information

Video S1 **This movie file shows the attacker chased the victim and hit it seven times, violently attacking and crushing the victim at the end of the movie.**
(MOV)

Video S2 **This movie file shows the attacker and the victim move independently and without any contact.**
(MOV)

Video S3 **This movie file shows another version of the aggressive interaction.**
(MOV)

Video S4 **This movie file shows two geometric figures interact in the same way as in Video S1, but the third, neutral figure moves independently.**
(MOV)
